# Supplementary material for: The role of structural connectivity on brain function through a Markov model of signal transmission
Source: bioRxiv. 2025 Feb 11:2024.11.10.622842. Preprint. [Version 2] doi: 10.1101/2024.11.10.622842 (PMC11844399; doi:10.1101/2024.11.10.622842)
Supplement: Supplementary file 1 [file NIHPP2024.11.10.622842V2-supplement-1.pdf]

bioRxiv preprint doi: https://doi.org/10.1101/2024.11.10.622842; this version posted February 11, 2025. The copyright holder for this preprint (which was not certified by peer review) is the author/funder, who has granted bioRxiv a license to display the preprint in perpetuity. It is made available under aCC-BY 4.0 International license.

Supplementary Information

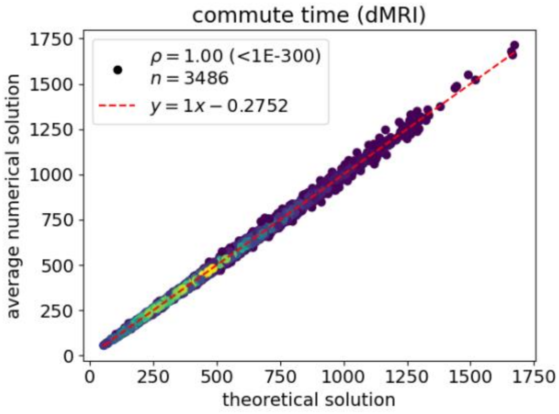

Figure S1: Theoretical expression for commute time (Equation 6) matches numerical results. *Commute time is calculated and simulated for a dMRI-derived structure from an arbitrary UK Biobank individual (subject id: 1000366) under the Desikan-Killiany atlas (84 brain regions). We run 1,000 replicate simulations for each pair of brain regions and take the mean number of steps to compare with the theoretical solution. Data points correspond to all possible pairs of brain regions (n = 84 x 83 / 2) and are colored to capture the density of points, with brighter colors corresponding to greater density. The symbol ρ corresponds to the Spearman correlation coefficient with its p-value following in parenthesis. The dashed red-line corresponds to the best-fit line. The equation is written in the legend where y corresponds to the average numerical solution; x corresponds to the theoretical solution.*

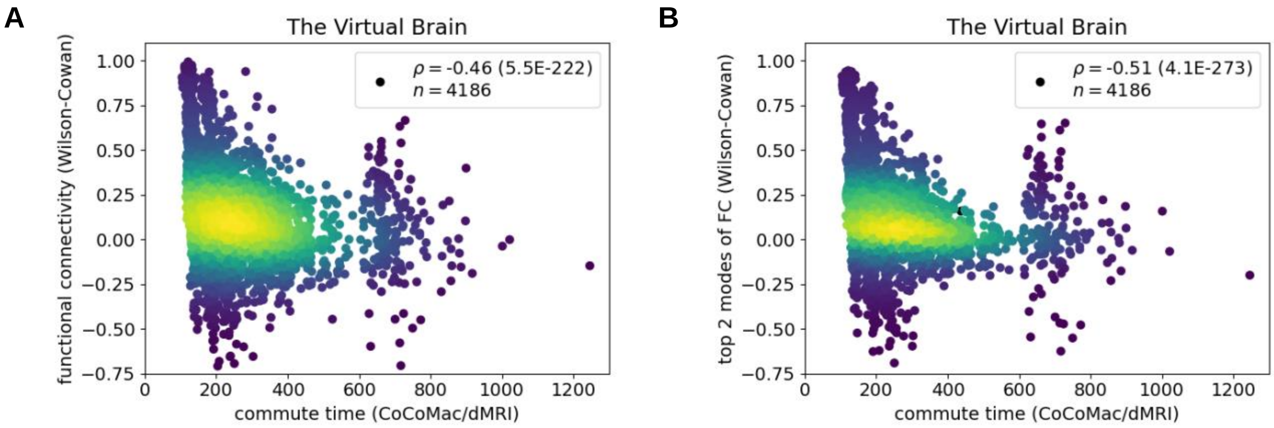

Figure S2: Commute time captures functional connectivity in a Wilson-Cowan simulation of neurosignaling. *The Wilson-Cowan simulation is run on connectivity data merged from the macaque brain (CoCoMac* (85, 86)*) and dMRI provided by The Virtual Brain* (37)*. Parameter values can be found in Table S1 and are chosen such that neuronal regions demonstrate stable oscillatory dynamics. Data points correspond to all possible pairs of brain regions and are colored to capture the density of points, with brighter colors corresponding to greater density. The same simulation is presented in both* ***A*** *and* ***B****; the difference is that only the result from the top two modes of the FC matrix is presented in* ***B****. For visual clarity, the x-axes are cutoff at a commute time value of 1300, however, there are a cluster of commute times at 4750 and they are included in correlation calculations and n.*

bioRxiv preprint doi: https://doi.org/10.1101/2024.11.10.622842; this version posted February 11, 2025. The copyright holder for this preprint (which was not certified by peer review) is the author/funder, who has granted bioRxiv a license to display the preprint in perpetuity. It is made available under aCC-BY 4.0 International license.

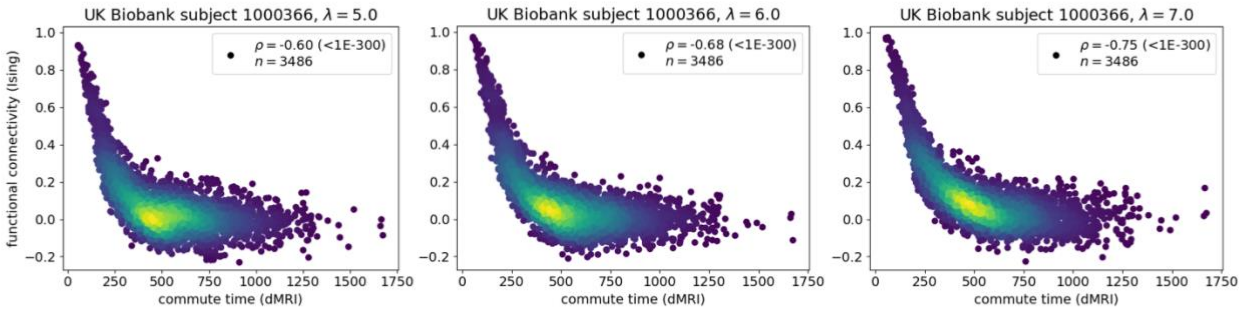

Figure S3: The commute time-functional connectivity correlation is mediated by the Ising model's coupling strength λ. *Three different Ising simulations are run on a dMRI-derived structure from the same UK Biobank individual (subject id: 1000366) for three different coupling strength values. The middle panel (λ = 6.0) is also displayed in Figure 2A.*

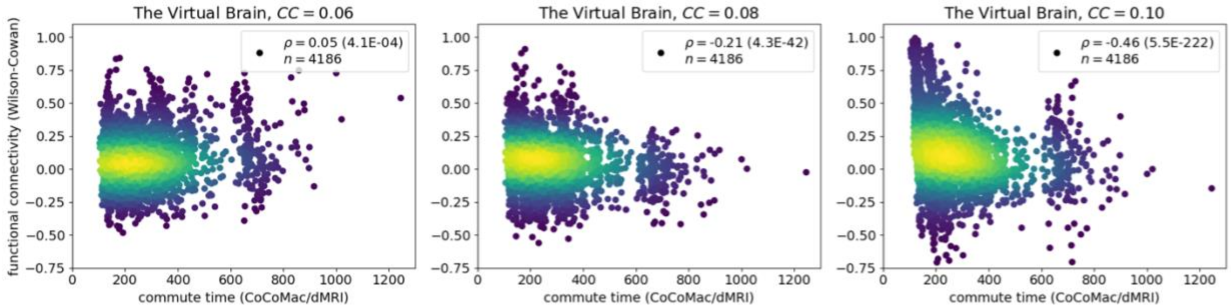

Figure S4: The commute time-functional connectivity correlation evaluated using the Wilson-Cowan model's global coupling constant (CC). *All other parameters are the same as in Figure S2 and can be found in Table S1. Three different Wilson-Cowan simulations are run on connectivity data merged from the macaque brain (CoCoMac* (85, 86)*) and dMRI provided by The Virtual Brain* (37) *for three different coupling constant values. For visual clarity, the x-axes are cutoff at a commute time value of 1300, however, there are a cluster of commute times at 4750 and they are included in correlation calculations and n. The right plot (CC = 0.10) is also displayed in Figure S2A.*

bioRxiv preprint doi: https://doi.org/10.1101/2024.11.10.622842; this version posted February 11, 2025. The copyright holder for this preprint (which was not certified by peer review) is the author/funder, who has granted bioRxiv a license to display the preprint in perpetuity. It is made available under aCC-BY 4.0 International license.

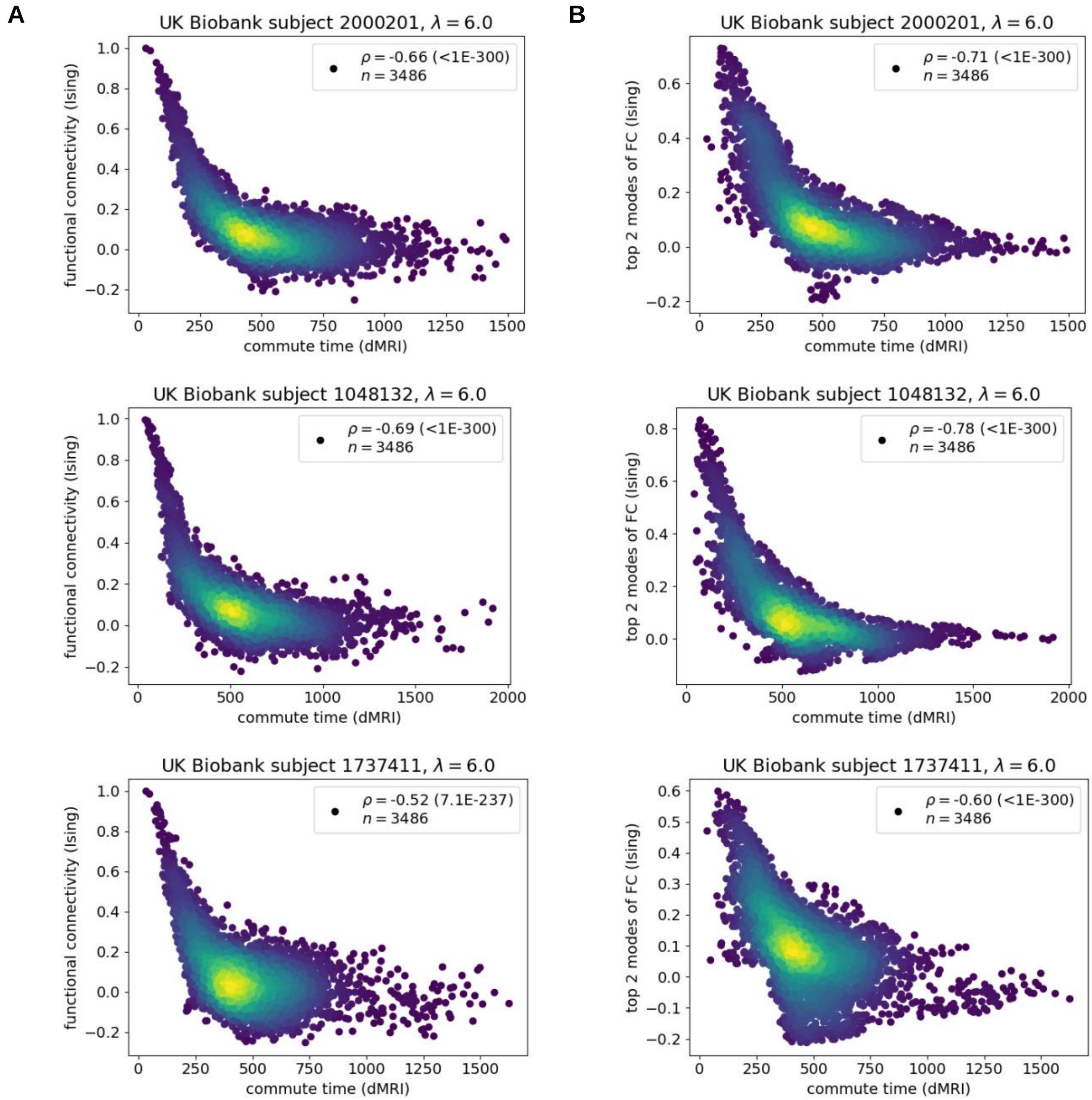

Figure S5: Commute time captures functional connectivity in mean-field Ising simulations for three different individuals' structures from the UK Biobank. *The same simulation is presented in each row of **A** and **B**; the difference is that only the top two modes of the FC matrix are included in **B**. Three sets of Ising simulations are performed on a dMRI-derived structure from three different UK Biobank individual (subject id: 2000201, 1048132, 1737411) under the Desikan-Killiany atlas (84 brain regions). The same global coupling strength parameter λ = 6.0 is employed for all individuals (Methods).*

bioRxiv preprint doi: https://doi.org/10.1101/2024.11.10.622842; this version posted February 11, 2025. The copyright holder for this preprint (which was not certified by peer review) is the author/funder, who has granted bioRxiv a license to display the preprint in perpetuity. It is made available under aCC-BY 4.0 International license.

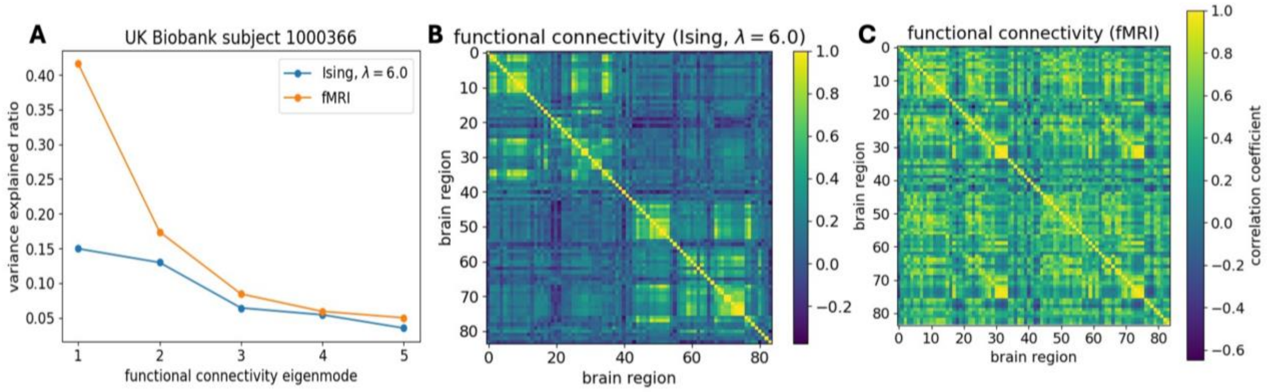

Figure S6: Comparison of the FC matrices based on Ising simulations and fMRI data. A. *The largest eigenvalues of the FC matrices from an Ising simulations on a dMRI-derived structure from subject 1000366 (blue curve) and the FC matrix from fMRI data of the same subject (orange curve). A coupling strength parameter λ = 6.0 is adopted under the Desikan-Killiany atlas (84 brain regions). FCs are the same as those presented in Figure 2A and in Figure 3A, respectively.* ***B-C.*** *The FC matrix derived from the Ising simulation with a coupling strength parameter λ = 6.0 on subject 1000366's dMRI structure under the Desikan-Killiany atlas (**B**) is qualitatively different than that of the FC matrix derived from fMRI data on the same individual under the same atlas (**C**).*

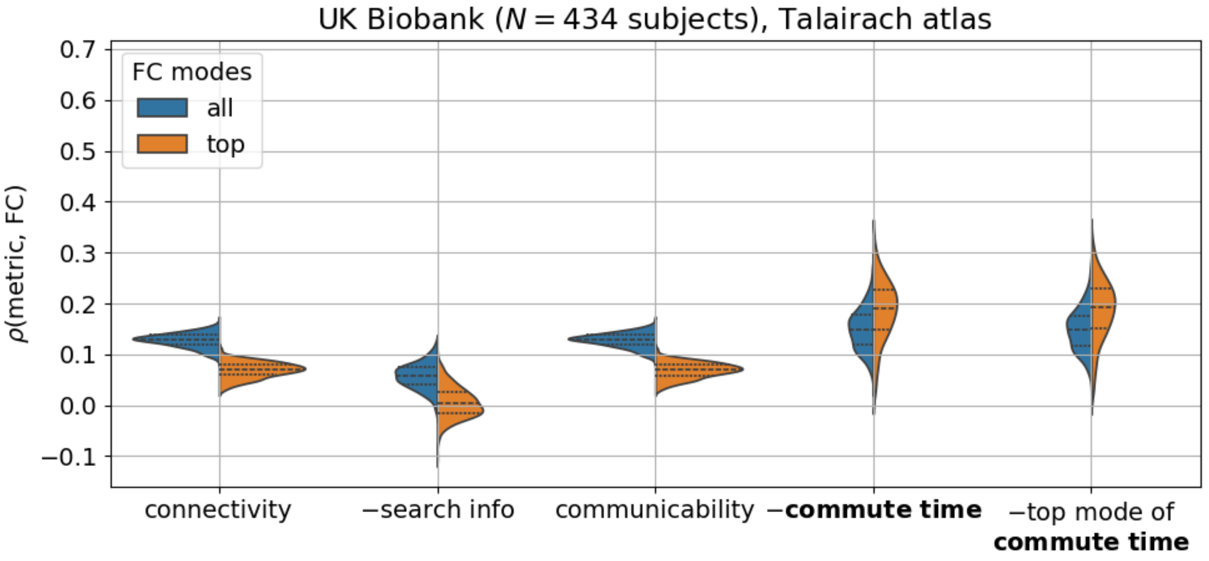

Figure S7: Commute time outperforms other structure-based metrics at predicting functional connectivities regardless of the brain atlas across the UK Biobank. *fMRI and dMRI data are processed according to the Talairach atlas (727 brain regions on average (Methods)). Corresponding Kolmogorov-Smirnov pairwise tests between metrics are summarized in Table S5.*

bioRxiv preprint doi: https://doi.org/10.1101/2024.11.10.622842; this version posted February 11, 2025. The copyright holder for this preprint (which was not certified by peer review) is the author/funder, who has granted bioRxiv a license to display the preprint in perpetuity. It is made available under aCC-BY 4.0 International license.

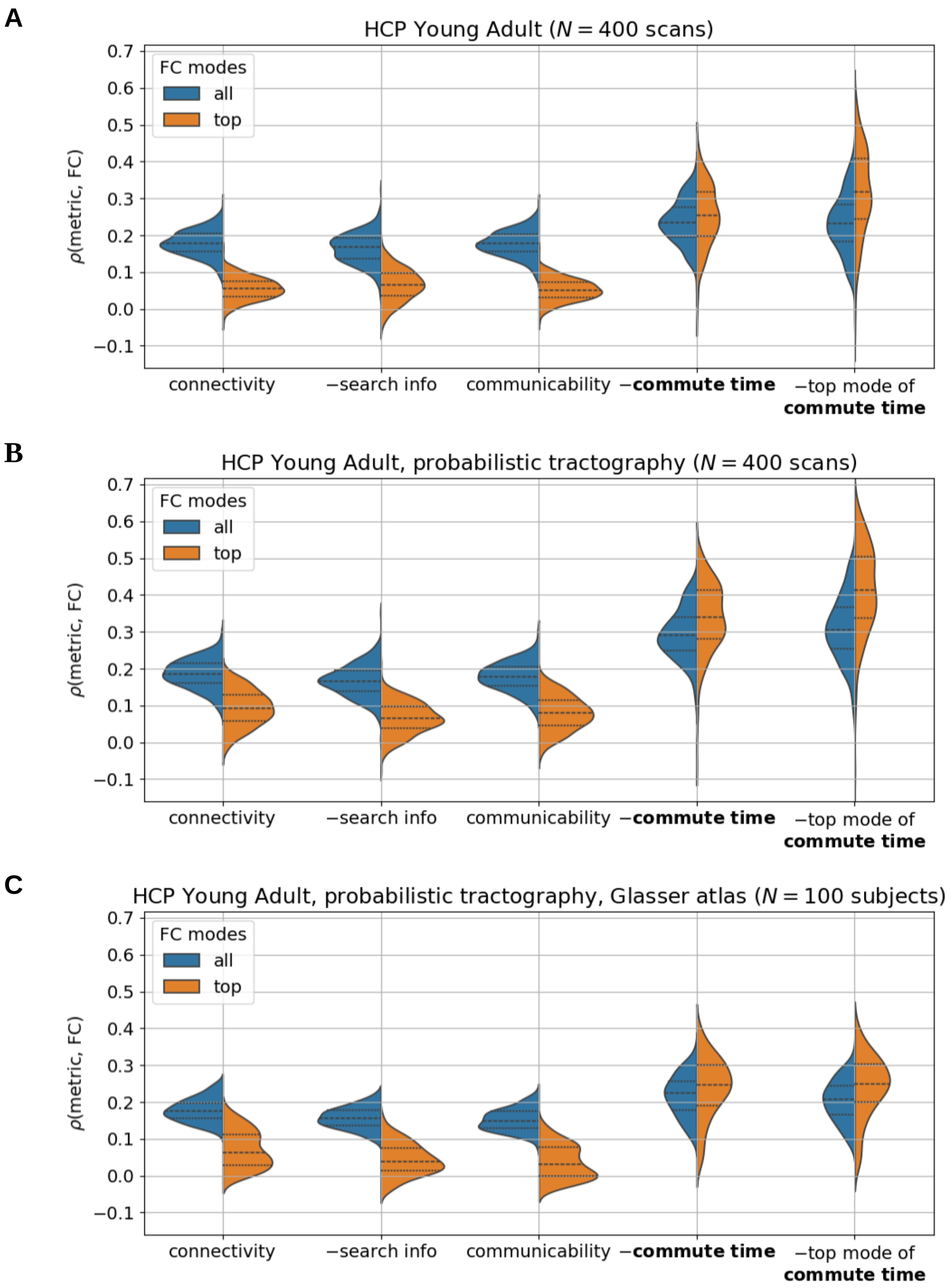

Figure S8: Commute time outperforms other structure-based metrics at predicting FCs for the Human Connectome Project (HCP) Young Adult dataset*. N corresponds to the number of brain scans considered from HCP Young Adult. Each subject had 4 brain scans and we consider individuals from the 100 Unrelated Subjects subset. fMRI and dMRI data are processed according to the Desikan-Killiany atlas. Commute time outperforms regardless of the tractography method (**A** is deterministic, **B-C** are probabilistic) or the atlas (**A-B** is the Desikan-Killiany atlas (84 brain regions),* ***C*** *is the Glasser atlas (360 brain regions)* (87)*. Results presented in* ***C*** *are taken from publicly data made available from another study* (16)*.*

bioRxiv preprint doi: https://doi.org/10.1101/2024.11.10.622842; this version posted February 11, 2025. The copyright holder for this preprint (which was not certified by peer review) is the author/funder, who has granted bioRxiv a license to display the preprint in perpetuity. It is made available under aCC-BY 4.0 International license.

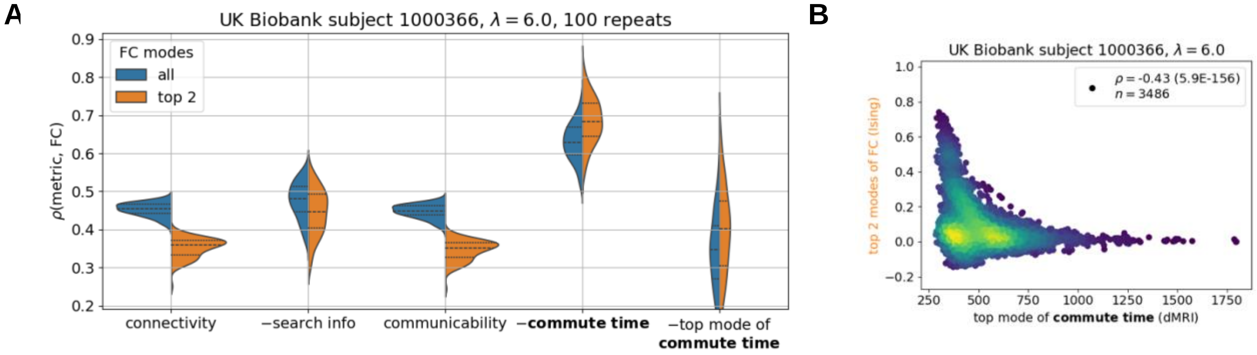

Figure S9: Top mode of commute time performs poorly for Ising simulations because function is entirely determined by the inputted structure. A. *is a reproduction of* ***Figure 2A*** *with an added column at the right corresponding to the top mode of commute time.* ***B.*** *Demonstrates a commute time-FC scatterplot across brain pairs when considering their respective top principal components (same simulation run as shown in* ***Figure 2A-B****). fMRI and dMRI data are processed according to the Desikan-Killiany atlas.*

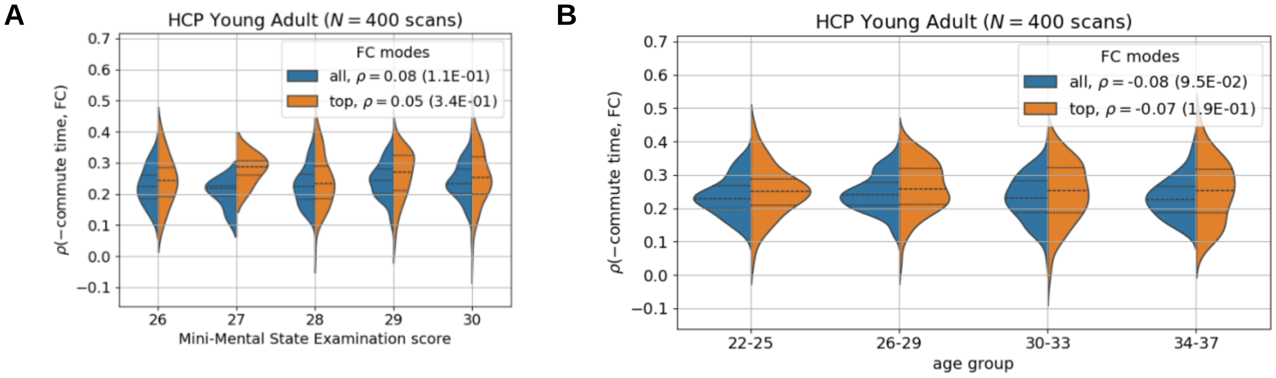

Figure S10: Minimal differences are seen between brain scans from different age groups (C), and different Mini-Mental State Examination scores (D) in the HCP Young Adult dataset. *fMRI and dMRI data are processed according to the Desikan-Killiany atlas.*

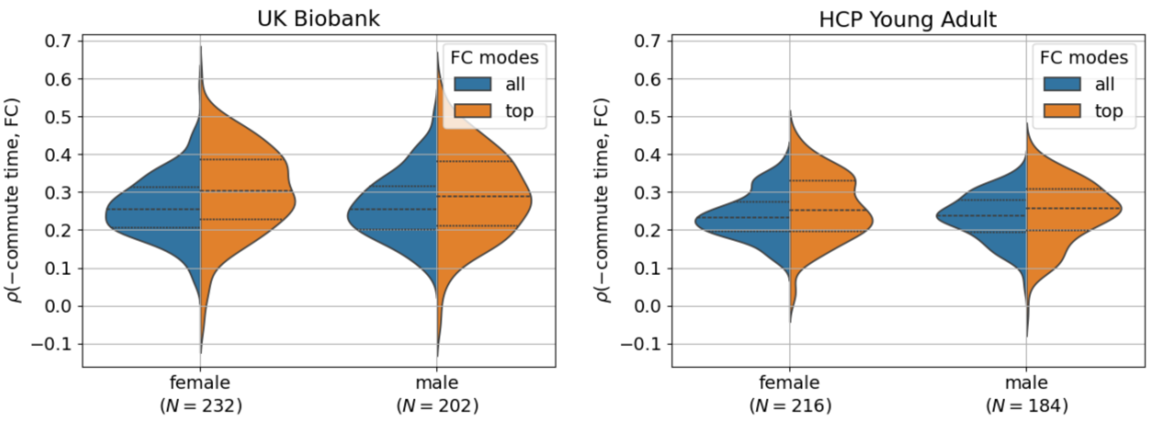

Figure S11: Minimal differences are seen in the commute time-FC correlation across brain scans from different sexes in the UK Biobank and HCP Young Adult datasets. *N corresponds to the number of brain scans considered for that particular group. fMRI and dMRI data are processed according to the Desikan-Killiany atlas.*

bioRxiv preprint doi: https://doi.org/10.1101/2024.11.10.622842; this version posted February 11, 2025. The copyright holder for this preprint (which was not certified by peer review) is the author/funder, who has granted bioRxiv a license to display the preprint in perpetuity. It is made available under aCC-BY 4.0 International license.

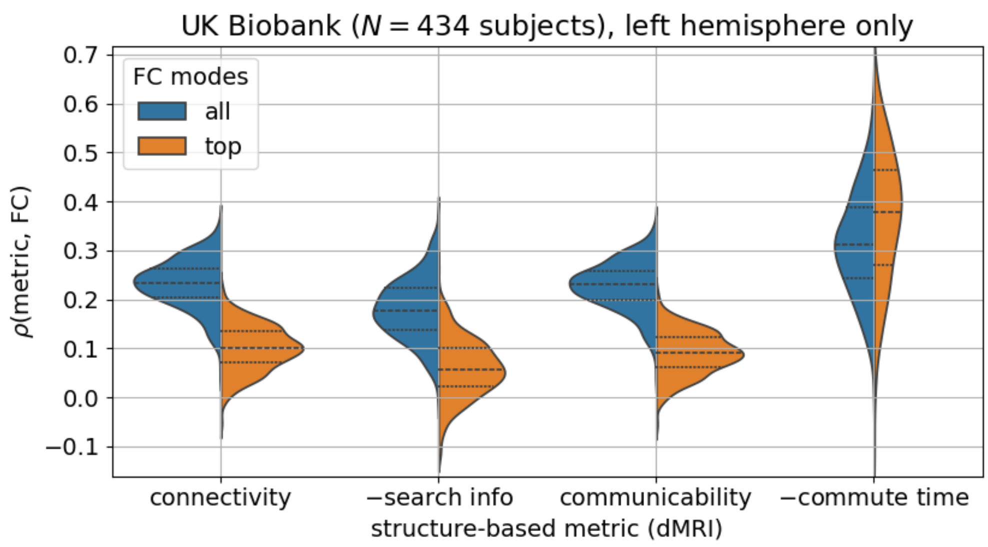

Figure S12: Commute time outperforms other structure-based metrics at predicting functional connectivities (FCs), even when only analyzing intra-hemisphere connections from the left hemisphere. *Corresponding Kolmogorov-Smirnov pairwise tests between metrics are summarized in Table S6. fMRI and dMRI data are processed according to the Desikan-Killiany atlas.*

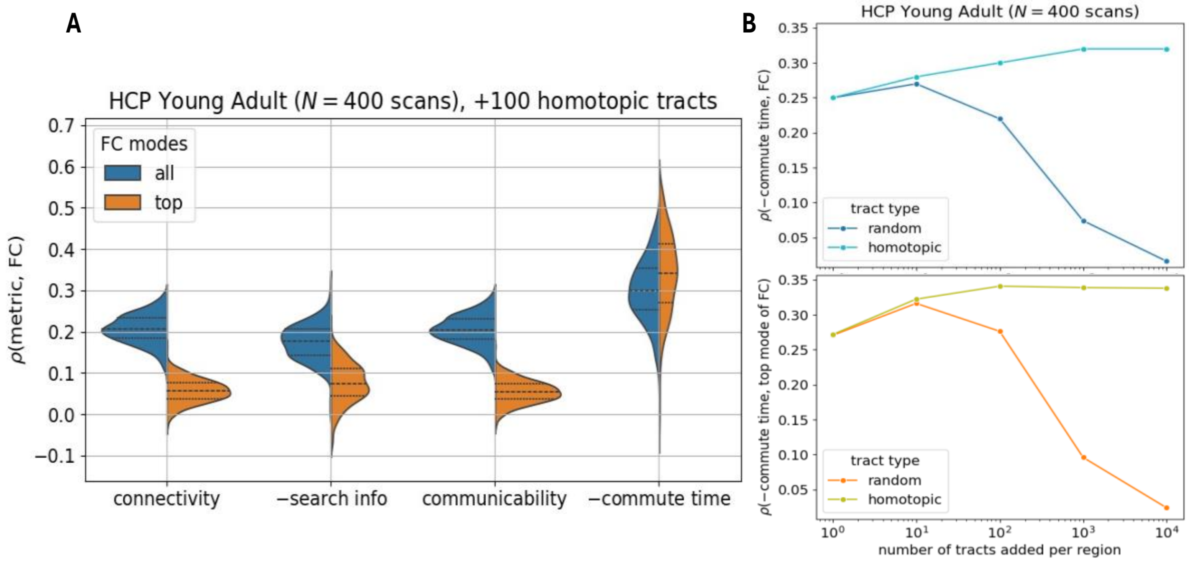

Figure S13: Effect of adding 100 homotopic connections per region between the two same regions across the brain hemispheres. A. *An improvement in structure-function correlations is observed, with commute time outperforming other structure-based metrics.* ***B.*** *White matter tracts added between homotopic regions improve the average commute time-FC correlation, especially compared to randomly added connections. The full distribution across all HCP Young Adult brain scans from the 100 Unrelated Subjects subset is shown for 100 added homotopic connections. Assessments are made for every order of magnitude up to 10,000 added white matter tracts. The entirety of the FC is considered in the top panel; only the top mode of the FC matrix is considered the bottom panel. fMRI and dMRI data are processed according to the Desikan-Killiany atlas.*

bioRxiv preprint doi: https://doi.org/10.1101/2024.11.10.622842; this version posted February 11, 2025. The copyright holder for this preprint (which was not certified by peer review) is the author/funder, who has granted bioRxiv a license to display the preprint in perpetuity. It is made available under aCC-BY 4.0 International license.

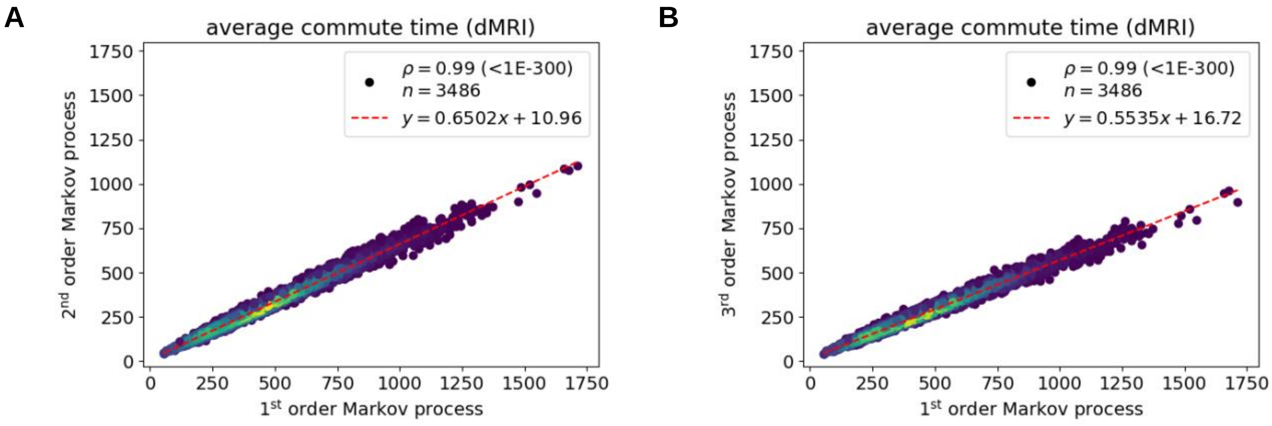

***Figure S14: Numerically calculating commute time with memory (2nd order and 3rd order Markov processes) strongly correlates with commute time without memory (1st order Markov process)**. The first order Markov process corresponds to the same simulation as in Figure S1 and has been simply called commute time throughout the text and SI. Second order and third order Markov processes are calculating by tweaking the 1st order simulation. For 2nd order, transition probabilities going back to the previous node along its path are set to zero. For example, if we have a network A-B-C, the following path is **not allowed** in a 2nd order Markov process: A→B**→A**→B→C. The subsequent rule is extended for a 3rd order Markov process for which the two previous nodes along its path are 'remembered.' All simulations are run on a dMRI-derived structure from an arbitrary UK Biobank individual (subject id: 1000366) under the Desikan-Killiany atlas. We run 1,000 replicate simulations for each pair of brain regions and plot the mean number of steps.*

Table S1: Wilson-Cowan model parameter descriptions and values for Figure S2 (*).

Parameter	Description	Value
Population time constants		
r_e	Excitatory refractory period [ms]	1 ms
r_i	Inhibitory refractory period [ms]	1 ms
k_e	Maximum value of the excitatory response function	1
k_i	Maximum value of the inhibitory response function	1
τ_e	Excitatory population, membrane time-constant [ms]	8 ms
τ_i	Inhibitory population, membrane time-constant [ms]	8 ms
Local network connectivity parameters		
c_{ee}	Excitatory to excitatory coupling coefficient	15.8
c_{ei}	Inhibitory to excitatory coupling coefficient	15.6
c_{ie}	Excitatory to inhibitory coupling coefficient	30
c_{ii}	Inhibitory to inhibitory coupling coefficient	6
α_e	Balance parameter between excitatory and inhibitory masses	1
α_i	Balance parameter between excitatory and inhibitory masses	1
Activation function parameters		
a_e	The slope parameter for the excitatory response function	1

bioRxiv preprint doi: https://doi.org/10.1101/2024.11.10.622842; this version posted February 11, 2025. The copyright holder for this preprint (which was not certified by peer review) is the author/funder, who has granted bioRxiv a license to display the preprint in perpetuity. It is made available under aCC-BY 4.0 International license.

a_i	The slope parameter for the inhibitory response function	1
b_e	Position of the maximum slope of the excitatory sigmoid function	0
b_i	Position of the maximum slope of the inhibitory sigmoid function	0
c_e	The amplitude parameter for the excitatory response function	1
c_i	The amplitude parameter for the inhibitory response function	1
ϑ_e	Excitatory threshold	0
ϑ_i	Inhibitory threshold	0
External perturbation parameters		
P	External stimulus to the excitatory population	-3.575
Q	External stimulus to the inhibitory population	-7.4
Global Connectivity parameters		
CV	Conduction velocity between brain regions	3.0
CC	Coupling constant between brain regions	0.1

(*) Descriptions are adapted from Tables 10 and 11 of Sanz-Leon et al. (37).

KS \\ p-value	connectivity	search info	communicability	commute time
**connectivity**	\\	1.0E-11 | 3.1E-31	0.11 | 0.21	2.2E-59 | 2.2E-59
**search info**	0.50 | 0.79	\\	3.4E-13 | 3.9E-33	2.6E-44 | 4.4E-55
**communicability**	0.17 | 0.15	0.53 | 0.81	\\	2.2E-59 | 2.2E-59
**commute time**	1.0 | 1.0	0.91 | 0.98	1.0 | 1.0	\\

Table S2: Summary statistics of Kolmogorov-Smirnov (KS) pairwise tests for distributions presented in Figure 2C when including all modes (left of the vertical line |) and only the top mode (right of |) of the functional connectivity matrix from Ising simulations on UK Biobank (subject id: 1000366) with a coupling strength parameter λ = 6. KS test statistics are entered in the bottom left of the table; corresponding p-values are in the top right.

KS \\ p-value	connectivity	search info	communicability	commute time
**connectivity**	\\	4.0E-18 | 5.1E-07	0.89 | 0.33	2.3E-70 | 1.1E-216
**search info**	0.30 | 0.19	\\	5.2E-16 | 2.4E-5	2.6E-84 | 3.3E-203
**communicability**	0.040 | 0.065	0.29 | 0.16	\\	1.5E-71 | 2.9E-218
**commute time**	0.59 | 0.95	0.64 | 0.93	0.59 | 0.95	\\

Table S3: Summary statistics of Kolmogorov-Smirnov (KS) pairwise tests for distributions presented in Figure 3C when including all modes (left of the vertical line |) and only the top mode (right of |) of the functional connectivity matrix from fMRI data of 434 UK Biobank individuals. KS test statistics are entered in the bottom left of the table; corresponding p-values are in the top right.

bioRxiv preprint doi: https://doi.org/10.1101/2024.11.10.622842; this version posted February 11, 2025. The copyright holder for this preprint (which was not certified by peer review) is the author/funder, who has granted bioRxiv a license to display the preprint in perpetuity. It is made available under aCC-BY 4.0 International license.

KS \\ p-value	mental disorder	brain disease	healthy
mental disorder	\\	0.15 | 0.35	0.12 | 0.27
brain disease	0.6 | 0.48	\\	0.78 | 0.32
healthy	0.55 | 0.46	0.16 | 0.24	\\

Table S4: Summary statistics of Kolmogorov-Smirnov (KS) pairwise tests for distributions presented in Figure 4A when including all modes (left of the vertical line |) and only the top mode (right of |) of the functional connectivity matrix from fMRI data of 434 UK Biobank individuals. KS test statistics are entered in the bottom left of the table; corresponding p-values are in the top right.

KS \\ p-value	connectivity	search info	communicability	commute time
connectivity	\\	3.3E-203 | 1.2E-152	1.0 | 1.0	2.9E-43 | 1.7E-184
search info	0.93 | 0.83	\\	1.2E-204 | 1.3E-151	8.5E-168 | 4.4E-206
communicability	0.014 | 0.028	0.93 | 0.83	\\	2.9E-43 | 1.7E-184
commute time	0.47 | 0.90	0.86 | 0.93	0.47 | 0.90	\\

Table S5: Summary statistics of Kolmogorov-Smirnov (KS) pairwise tests for distributions presented in Figure S7 when including all modes (left of the vertical line |) and only the top mode (right of |) of the functional connectivity matrix from fMRI data under the Talairach atlas of 434 UK Biobank individuals. KS test statistics are entered in the bottom left of the table; corresponding p-values are in the top right.

KS \\ p-value	connectivity	search info	communicability	commute time
connectivity	\\	1.1E-32 | 1.5E-21	0.47 | 0.0098	1.7E-45 | 1.4E-173
search info	0.41 | 0.33	\\	5.5E-28 | 2.9E-16	2.8E-78 | 6.2E-176
communicability	0.058 | 0.11	0.38 | 0.29	\\	2.9E-48 | 3.0E-183
commute time	0.48 | 0.88	0.62 | 0.88	0.49 | 0.89	\\

Table S6: Summary statistics of Kolmogorov-Smirnov (KS) pairwise tests for distributions presented in Figure S12 when including all modes (left of the vertical line |) and only the top mode (right of |) of the functional connectivity matrix from fMRI data for only left hemisphere regions of 434 UK Biobank individuals. KS test statistics are entered in the bottom left of the table; corresponding p-values are in the top right.

Dataset	age range	<age>± std(age)	sex
UK Biobank	46-79	62.6 ± 7.4	232F / 202M
HCP Young Adult	22-36	29.1 ± 3.7	54F / 46M

Table S7: Demographic information of the individuals considered in this paper. Ages are in units of years.

bioRxiv preprint doi: https://doi.org/10.1101/2024.11.10.622842; this version posted February 11, 2025. The copyright holder for this preprint (which was not certified by peer review) is the author/funder, who has granted bioRxiv a license to display the preprint in perpetuity. It is made available under aCC-BY 4.0 International license.

Dataset	field	repetition	echo	flip angle	voxel size	total time
**UK Biobank**	3T	735 ms	39 ms	52°	2.4x2.4x2.4	490
**HCP Young**	3T	720 ms	33.1 ms	52°	2x2x2 mm^3	1200

Table S8: Functional MRI acquisition parameters of the datasets.

Commute time derivation

We expand upon the derivation presented in the Methods by illustrating how applying Greville's formula twice takes us from Equation 10 and Equation 11. If $\boldsymbol{A}_k$ is a m x k matrix, Greville's formula states that the generalized matrix of $\boldsymbol{A}_k$ by appending an additional column a_{k+1} ($\boldsymbol{A}_{k+1}^{+}$) can be expressed as (68, 69),

Equation 15

$$\boldsymbol{A}_{k+1}^{+} = [\boldsymbol{A}_k, a_{k+1}]^{+} = \begin{bmatrix} \boldsymbol{A}_k^{+} - d_k b_k \\ b_k \end{bmatrix}$$

The symbols d_k and b_k are defined as,

Equation 16

$$d_k = \boldsymbol{A}_k^{+} a_{k+1},\; c_k = a_{k+1} - \boldsymbol{A}_k d_k$$
$$b_k = \begin{cases} c_k^T, & \text{if } c_k \neq 0 \\ \left(1 + d_k^T d_k\right)^{-1} d_k^T \boldsymbol{A}_k^{+}, & \text{if } c_k = 0 \end{cases}$$

With Greville's formula, we can express $\widehat{\boldsymbol{\Gamma}}^{-1}$ in terms of $\boldsymbol{\Gamma}^{-1}$ as follows. First, we define $\widetilde{\boldsymbol{\Gamma}}$ as an (n-1) x n matrix.

Equation 17

$$\boldsymbol{\Gamma} = \begin{bmatrix} \widetilde{\boldsymbol{\Gamma}} \\ r^T \end{bmatrix}$$

It can be shown that the row vector $r^T = -\widehat{\mathbf{1}}^T \widetilde{\boldsymbol{\Gamma}}$ ensures the condition that the sum of rows of the Laplacian matrix is equal to zero ($\mathbf{1}^T \boldsymbol{\Gamma} = \mathbf{0}$).

To connect $\widetilde{\boldsymbol{\Gamma}}$ to $\widehat{\boldsymbol{\Gamma}}$, we have the following relationship:

Equation 18

$$\widetilde{\boldsymbol{\Gamma}} = [\widehat{\boldsymbol{\Gamma}}, s]$$

It can be shown that the vector $s = -\widehat{\mathbf{1}}^T \widetilde{\boldsymbol{\Gamma}}$ ensures that the sum of columns of the Laplacian matrix is equal to 0 ($\boldsymbol{\Gamma}\mathbf{1} = \mathbf{0}^T$).

We are now ready to apply Greville's formula. We first calculate the pseudoinverse of $\widetilde{\boldsymbol{\Gamma}}$,

Equation 19

$$\widetilde{\boldsymbol{\Gamma}}^{-1} = \begin{bmatrix} \widehat{\boldsymbol{\Gamma}}^{-1} + \widehat{\mathbf{1}} b_k \\ b_k \end{bmatrix}$$

bioRxiv preprint doi: https://doi.org/10.1101/2024.11.10.622842; this version posted February 11, 2025. The copyright holder for this preprint (which was not certified by peer review) is the author/funder, who has granted bioRxiv a license to display the preprint in perpetuity. It is made available under aCC-BY 4.0 International license.

We do not need to directly evaluate b_k from Equation 16. Instead, we notice that b_k must be equal to the removed j^{th} row in $\widehat{\boldsymbol{\Gamma}}$ that is present in $\widetilde{\boldsymbol{\Gamma}}$.

Equation 20

$$\widetilde{\boldsymbol{\Gamma}}^{-1} = \begin{bmatrix} \widehat{\boldsymbol{\Gamma}}^{-1} \\ 0 \end{bmatrix} + \begin{bmatrix} \widehat{\mathbf{1}} b_k \\ b_k \end{bmatrix}$$
$$\rightarrow b_k = \left[\widetilde{\boldsymbol{\Gamma}}^{-1}\right]_j$$

In component form, Equation 20 is equivalent to:

Equation 21

$$\left[\widetilde{\boldsymbol{\Gamma}}^{-1}\right]_{lm} = \left[\widehat{\boldsymbol{\Gamma}}^{-1}\right]_{lm} + \left[\widetilde{\boldsymbol{\Gamma}}^{-1}\right]_{jm}$$
$$\rightarrow \left[\widehat{\boldsymbol{\Gamma}}^{-1}\right]_{lm} = \left[\widetilde{\boldsymbol{\Gamma}}^{-1}\right]_{lm} - \left[\widetilde{\boldsymbol{\Gamma}}^{-1}\right]_{jm}$$

We can perform the same steps as in Equation 19 and Equation 20 to express $\widetilde{\boldsymbol{\Gamma}}^{-1}$ in terms of $\boldsymbol{\Gamma}^{-1}$,

Equation 22

$$\left[\widetilde{\boldsymbol{\Gamma}}^{-1}\right]_{no} = \left[\boldsymbol{\Gamma}^{-1}\right]_{no} - \left[\boldsymbol{\Gamma}^{-1}\right]_{jn}$$

Inserting Equation 22 into Equation 21, we finally have our expression for $\widehat{\boldsymbol{\Gamma}}^{-1}$ in terms of $\boldsymbol{\Gamma}^{-1}$.

Equation 23

$$\left[\widehat{\boldsymbol{\Gamma}}^{-1}\right]_{lm} = \left[\boldsymbol{\Gamma}^{-1}\right]_{lm} - \left[\boldsymbol{\Gamma}^{-1}\right]_{jl} - \left[\boldsymbol{\Gamma}^{-1}\right]_{jn} + \left[\boldsymbol{\Gamma}^{-1}\right]_{jj}$$

Equation 23 is inserted in Equation 10 to obtain Equation 11.
